# Differences between living and deceased donation in human uterus transplantation: A narrative review

**DOI:** 10.18502/ijrm.v21i3.13195

**Published:** 2023-04-14

**Authors:** Sakineh Taherkhani

**Affiliations:** School of Medicine, Arak University of Medical Sciences, Arak, Iran.

**Keywords:** Living donors, Deceased donors, Brain death donors, Uterus transplantation, Absolute uterine factor infertility, Review.

## Abstract

The decision to use a living or deceased donor to perform uterus transplantation (UTx) is an evaluation of benefit and harm and is based on the medical team's choices. The current study determines the differences between living and deceased donation in human UTx according to determinant factors in choosing the donor type. For this review study, the PubMed database was searched without time, language, and location limitations up to May 2022. From 113 identified articles, 45 papers were included in the study for review. According to the results, in comparison to living donation, the biggest advantage of deceased donation is the lack of surgical and or psychological risks for the donor. In contrast, a comprehensive pre-transplantation medical assessment is less possible in deceased donation, and preplanned surgery cannot be realized. According to published peer-reviewed clinical trials on UTx, the graft failure rates in living and deceased donor UTx are 21% and 36%, respectively. Supposing all recipients who did not have graft failure underwent embryo transfer, live birth rates in living and deceased donor UTx procedures are almost 63% and 71%, respectively. Currently, considering the occurrence of live births from both donations, particularly from nulliparous deceased donor, increased demand for UTx in the near future, shortage of uterus grafts, and lack of sufficient data for a comprehensive comparison between the 2 types of donation, the use of both donations still seems necessary and rational.

## 1. Introduction

In the literature, uterus transplantation (UTx) is considered the latest solid organ transplantation (1), which involves taking a uterus graft from a donor and transplanting it to another woman (2). This novel approach to infertility treatment includes using both fields of assisted reproduction and reproductive surgery (3). UTx is the only treatment for congenital or acquired absolute uterine factor infertility but has not been recognized as a standard method (1). Women without a uterus due to congenital (e.g., uterine agenesis in Mayer-Rokitansky-Küster-Hauser syndrome) or iatrogenic (e.g., hysterectomy following cancer or refractory bleeding) etiologies or women with a malfunctioning uterus due to intrauterine pathologies (e.g., severe adenomyosis, intrauterine adhesions like Ashermann syndrome, congenital uterine malformation) are classified as patients with absolute uterine factor infertility (4, 5). Formerly, these women had no alternatives but adoption and gestational surrogacy to experience motherhood (6). Currently, UTx is the only therapeutic intervention that can restore these women's reproductive function and anatomy (7).

### Origin of uterus graft in UTx 

The UTx is carried out with uteri from a living donor (LD) and deceased donor (DD); the latter also points out a brain-dead/multiorgan donor (3). The LD is usually a family member or a friend (2). The LD can be genetically related to the recipient, such as her mother (8). The UTx has also been conducted via nondirected LDs, such as women volunteering for hysterectomy (e.g., in female-to-male sex reassignment surgery in transgenders) (2) or those who are candidates for hysterectomy due to benign causes (9, 10). In deceased donation, the uterus graft is obtained from a brain-dead woman (11). When no related or suitable donor is available, the deceased donation is the only potential source for a uterus graft (8). Deceased donation is more ethically approved due to possible harm to the LD and concerns about the LD's consent and the possibility of her regret (4, 12-14).

### History of UTx

The first human UTx was carried out in Saudi Arabia from an LD in April 2000, although it did not lead to live birth (9). Less than 15 yr later, the first live birth worldwide following LD UTx was recognized in Sweden in September 2014 (15). Almost 3 yr later, the first live birth worldwide following DD UTx was reported in Brazil in December 2017 (16). Table I shows an overview of the published peer reviewed UTx clinical trials, although Brännström et al. believe that numerous cases have not yet been published (3).

There are still discussions and controversies surrounding the type of donor for UTx (2), as LD UTx is a very invasive surgery, and on the other hand, according to the organ transplantation guidelines, the organ should be harvested from a DD (17). The decision to use LD or DD is a benefit and harm assessment and is based on the medical team's choices (18). The present study aims to determine the differences between living and deceased donation in human UTx based on determinant factors in choosing the donor type.

**Table 1:** Summary of UTx procedures published based on the characteristics of the donor, the recipient, and the outcomes of the transplant


gray!50 **Author, Yr (Ref)**	**Country (Number of reported UTx)**	**Type of donor**	**Age of donor (Yr)**	**Age of recipient (Yr)**	**Indication for UTx (Number)**	**Graft failure (Number)**	**Number of live birth (GA at birth, wk + days)**
**Fageeh ** ***et al.*** **, 2002 (9)**	Saudi Arabia (1)	LD	46	26	Hysterectomy for PPH	Yes (1). 1 Hysterectomy for acute vascular thrombosis on POD 99	N/A
**Akar ** ***et al.*** **, 2013 (19)**				
**Ozkan ** ***et al.*** **, 2022 (20)**	Turkey (1)	DD	22	23	MRKHs	No	1 (28)
**Brännström ** ***et al.*** **, 2015 (15)**											
**Brännström ** ***et al.*** **, 2016 (21)**				
**Brännström ** ***et al.*** **, 2014 (22)**				
**Broecker ** ***et al.*** **, 2021 (23)**	Sweden (9)	LD	M = 53 ± 7	M = 31.5 ± 3.9	MRKHs (8)
Radical					
hysterectomy					
for cervical					
cancer (1)	Yes (2). 1 hysterectomy for
acute bilateral thrombotic	
uterine artery occlusions on	
POD 3. 1 hysterectomy for	
persistent intrauterine
infection on POD 105	9 (31+5, 34+6) ${}^{*}$
**Huang ** ***et al.*** **, 2020 (24)**					
**Wei ** ***et al.*** **, 2017 (25)**		China (1)	LD	42	22	MRKHs	No	1 (33+6)
**Flyckt ** ***et al.*** **, 2016 (11)**												
**Flyckt ** ***et al.*** **, 2017 (26)**	United States (1)	DD	N/R	N/R	MRKHs	Yes (1). 1 hysterectomy
for severe candida						
infection of the
graft on POD 12	N/A
**Ejzenberg ** ***et al.*** **, 2018 (16)**		Brazil (1)	DD	45	32	MRKHs	No	1 (35+3)
**Puntambekar ** ***et al.*** **, 2019 (27)**				
**Puntambekar ** ***et al.*** **, 2018 (28)**	India (4)	LD	M = 45.5	M = 25.2	MRKHs (4)	N/R	N/R
**Flyckt ** ***et al.*** **, 2020 (29)**								United States (1)	DD	24	35	MRKHs	No	1 (34+2)
**Brucker ** ***et al.*** **, 2020 (30)**	Germany (4)	LD	M = 45	M = 28.2	MRKHs (4)	N/R	2 (35+1, 36+3)
**Brännström ** ***et al.*** **, 2020 (31)**				
**Brännström ** ***et al.*** **, 2020 (32)**	Sweden (8)	LD	M = 49.8	M = 28	MRKHs (8)	Yes (2). 2 hysterectomy for
uterine necrosis at about						
POM 1 and 6	1 (36+1)
**Johannesson ** ***et al.*** **, 2021 (33)**					
**Testa ** ***et al.*** **, 2020 (34)**	United States
(20)		18 LD
2 DD	M = 37.7		M = 28.9	MRKHs (18)
hysterectomy				
for benign
disease (2)	Yes (6). 6 hysterectomy
(5 LD UTx and 1 DD	
UTx) between POD 0 and 9
Causes include vascular
complications, irreversible
ischemic damage, and
graft failure for
appropriate reperfuse
despite patent vasculature	11 from LD
UTx and 1	
from DD UTx
(Median GA,
36+6/7
range GA,
30+6/7-38)
**Fronek ** ***et al.*** **, 2021 (35)**	Czech Republic (10)	5 LD 5 DD	46 ± 14	28 ± 3	MRKHs (10)	Yes (3). 1 hysterectomy for arterial thrombosis on POD 7 in DD UTx 1 hysterectomy for venus thrombosis on POD 15 in LD UTx 1 hysterectomy for HSV infection and protracted rejection on POD 213 in DD UTx	2 from LD UTx (36+2, 35+3) and 1 from DD UTx (34+6)
**Carmona ** ***et al.*** **, 2021 (36)**	Spain (1)	LD	39	31	MRKHs	N/R	N/R
**Vieira ** ***et al.*** **, 2021 (37)**	Brazil (1)	LD	50	33	MRKHs	N/R	N/R
UTx: Uterus transplantation, LD: Living donor, DD: Deceased donor, GA: Gestational age, PPH: Post-partum hemorrhage, POD: Postoperative day, POM: Postoperative month, N/A: Not applicable, N/R: Not reported, MRKHs: Mayer-Rokitansky-Kuster-Hauser syndrome, M: Mean, HSV: Herpes simplex virus. *Gestational age was not reported for other cases							

## 2. Materials and Methods

### Search strategy

In this narrative review study, for the identification of the related articles, the PubMed database was searched using the terms “uterus transplantation” or “uterine transplantation” in combination with the associated terms, such as “living donor”, “deceased donor”, and “brain-dead donor”, up to 10 February 2022 without time, location, and language limitations. The details of the search strategy used are presented as follows:

1: “Uterine transplantation” [Text Word] OR “Uterine transplantation” [MeSH Terms]

2: “Uterus transplantation” [Text Word] OR “Uterus transplantation” [MeSH Terms]

3: 1 OR 2

4: “living donor” [Text Word] OR “living donor” [MeSH Terms]

5: “live donor” [Text Word] OR “live donor” [MeSH Terms]

6: “living donation” [Text Word] OR “living donation” [MeSH Terms]

7: “brain dead donor” [Text Word] OR “brain dead donor” [MeSH Terms]

8: “deceased donor” [Text Word] OR “deceased donor” [MeSH Terms]

9: “deceased donation” [Text Word] OR “deceased donation” [MeSH Terms]

10: “multiorgan donor” [Text Word] OR “multiorgan donor” [MeSH Terms]

11: 4 OR 5 OR 6 OR 7 OR 8 OR 9 OR 10

12: 3 AND 11

On May 1, 2022, the search results were updated. In addition to the electronic search, a manual search was conducted by reviewing the reference lists of the retrieved articles to identify more related articles.

### Inclusion and exclusion criteria

All types of articles (such as original studies, reviews, case reports, letters, and commentaries) with a focus on the features associated with human UTx using LD or DD, including the risks and advantages for donor and recipient, the evaluation of the donor, the procurement of the uterine, the implementation of the transplantation procedure (logistics of surgical procedure, waiting time, and transplantation costs), and the outcomes of the transplantation (graft failure and live birth) were included in the study. Nonhuman UTx studies were excluded from this study.

### Study selection and data extraction

The retrieved articles were imported into the Endnote software. For selecting articles, first, the titles and abstracts of the papers were screened, and unrelated articles and related ones without available full-text versions were excluded. For the data extraction, the full texts of the relevant articles identified in the previous stage were reviewed, and the data related to the above-mentioned features were extracted.

## 3. Results

A total of 113 English articles were retrieved from the PubMed database. After excluding unrelated articles and those without available full-text versions, the full texts of 45 articles were reviewed. The results of the study, including the differences between living and deceased donation regarding the risks and advantages of the UTx for the donor and recipient, the evaluation of the donor, the procurement of the uterine, the implementation of the transplantation procedure, and the outcomes of the transplantation, will be discussed in the subsequent sections.

### Risks of the UTx 

#### Physical risks for the donor

As the uterus is not a vital organ, only slight harm to LD is acceptable (30); however, uterus retrieval from an LD is generally a high-risk, long, complex (11), and invasive surgery (17). The risks of hysterectomy in LDs are higher than in conventional hysterectomy (2). The main intraoperative and postoperative complications include urinary tract complications, infection, bleeding, thrombosis, and hematoma (38). Bowel injuries, vaginal cuff dehiscence, ureterovaginal fistula, urinary tract infection, nocturia, leg pain, fecal impaction, meralgia paresthetica (18), and injury to the ureter (4), the bladder, the rectum, the pelvic nerves, and the iliac vessels (39) are other reported complications. Several studies have demonstrated that more than 1 in 10 LDs develop complications requiring further surgical intervention following uterine donation (7).

If the ovaries of a premenopausal LD are removed during uterus procurement surgery (25) or ovarian veins are used for vascular anastomosis (which can lead to the disruption of ovarian blood flow and excision of the ovaries), the LD might be at risk for early menopause (28, 40) and its aftermaths, such as an increased risk of mortality and morbidity (6). The reason is that ovaries are vital endocrine organs that can play protective roles in women even decades after menopause, and on the other hand, the exogenous hormone therapy used to reduce symptoms in menopausal women is associated with long-term health risks (41).

The prolonged duration of LD surgery in UTx is associated with an increased risk of thromboembolic events, especially pulmonary embolism (8). Furthermore, in some studies, for 3 months before the operation, hormone replacement therapy for menopausal LDs has been prescribed to ensure uterine menstrual functionality and possibly increase the circulation of uterine arteries (42). This approach also puts LDs at an increased risk of thromboembolic events before and immediately after the operation (43).

Nevertheless, in clinical trials performed, surgery complications in LDs have been identified and successfully resolved (29); on the other hand, increasing experience in performing UTx procedures and using less invasive techniques, such as robotic-assisted laparoscopy, the rate, and severity of surgical complications for LDs have decreased (30); however, the long-term risks, particularly in LDs that have undergone oophorectomy, have not yet been fully elucidated (29).

#### Physical risks for the recipient

Uterine recipients of DDs might be at risk for hemorrhage. This is because the dissection and ligation of the uterus vessels of DDs are conducted outside the body during the back-table preparation stage and in this situation, small vessels are difficult to find around a white uterus that has been flushed with a preservative solution. This makes vascular assessment before the reperfusion more difficult in the uterus of DDs in comparison to LDs. If the microscopic vessels of the tissues adjacent to the uterus are not meticulously ligated for transplantation, hemorrhage after reperfusion will occur during recipient surgery, and if homeostasis is not maintained, the recipient will develop intra-abdominal complications, hematoma surrounding the uterus, and retroperitoneal hematoma (17).

#### Psychological risks for the donor

Unfavorable psychological consequences in LDs might occur in some conditions, such as the early failure of the graft, the breaking down of the donor-recipient relationship, and if the transplanted uterus does not lead to the delivery of a healthy newborn despite surviving for a long time (8). Kisu et al. believe that unstable mental conditions, such as anxiety and depression, might occur even with favorable outcomes (40). The LDs might feel loss or harm, as numerous women have such feelings following a hysterectomy (18). Although living without a uterus is clinically insignificant, there are important consequences for uterine donation. Since the uterus symbolizes sexuality, femininity, attractiveness, vitality, youth, and childbearing; hysterectomy can lead to postoperative regression, loss of feminine self-image, distortion of body image (44), decreased quality of life (39, 44-48), loss of gender identity (13, 49), changes in sexual libido (39), increased sexual dysfunction (13, 48-50), and decreased sexual satisfaction (49). Surgical scars on the abdomen can distort body image, and numerous women might feel physically unattractive following the operation (40).

#### Psychological risks for the recipient

For the recipient, receiving a uterus from an LD can be an unpayable debt (4, 51). The complex relationship between LD and the recipient might lead to embarrassment, anxiety, and guilt in the recipient due to the involvement of another healthy woman in her misfortune (49). In the case of using relative LDs, both the donors and the recipients might have concerns regarding family dynamics and experience external and internal pressures for donation or reception of the uterus. Therefore, recipients might prefer DD to avoid or decrease worries related to consent, voluntariness, and risks for LD (52). LDs, such as mothers, sisters, and aunts, sometimes claim a right to children born from donated uteri and expect more contact with the newborns, similar to the expectation of surrogate mothers (53). Consequently, exerting control of an LD over the recipient in the future or influencing her decisions can occur in cases of LD UTx (50).

### Advantages of the UTx

#### Psychological advantages for the donor

The promotion of psychological well-being due to helping an individual for giving birth is one of the psychological benefits of donation for LD (18). The LDs experience a long-term increase in self-esteem, happiness, and quality of life following donation and feel grateful for having the opportunity to live with a loved one who regains her health. Particularly, the family gains considerable psychological benefit from the donation (53). If a successful delivery occurs following a donation between related individuals, the positive effect of having a healthy child on quality of life and psychological health might last for many years (4). Moreover, depending on the nature of their relationship with the recipients, LDs might enjoy having a relationship with the born children (53).

### Evaluation of the donor

In LD UTx, there is an opportunity for long term and meticulous evaluation of LD and her uterus before donation (8, 54). These evaluations increase the possibility of transplanting a uterus with a high chance of survival after transplantation and a high probability of pregnancy and childbirth. Detailed and precise obstetrics and gynecology history with a comprehensive medical history can be obtained from the LD that can assist in deciding on the exclusion of potential donors with a risk of repeated miscarriages, preterm birth, preeclampsia, and other high-risk obstetric conditions. Periodic prescription of estrogen-gestagens can be used to assess uterine bleeding patterns and thickness, as well as the appearance of the endometrium in postmenopausal LDs (8).

In deceased donation, there might be limited information about the medical history of DD, which can have undesirable consequences for the transplantation. In multiorgan donation in DD, there is often less than 24 hr since the time of consent to the removal of organs (8). In this situation, the results of all tests should be available within 12-24 hr (3). This short time might reduce the likelihood of routine pretransplantation assessments and evaluation of uterine arteries (8). In addition, it is not possible to prescribe hormone replacement therapy for postmenopausal DDs (55).

Examining psychological factors, including psychological stability, is an important issue only in LD screening (3). The donor should not have poor psychological health or psychiatric disorders (8); in other words, she should have the mental capacity to consent (56). The donor should be briefed about donation's short-term and long-term risks (41), and her informed and valid consent should be obtained by physicians and psychologists after in-depth and compulsory consultation (10). In the case of deceased donation, the DD (before death) or her surrogate (after death) should be consented (53).

### Procurement of uterine

The technique of uterus retrieval from a DD is like that of an LD but is simpler and faster (8, 17). A Turkish team has reported the length of the procurement of the uterus from a DD as 2 hr (57), and in a trial in the United States, the authors have reported this time as approximately 60-90 min (11). In the first UTx trial by a Swedish team that included 9 LDs, the donors' surgery duration was reported as 10-13 hr (3). Gradually, the duration of uterus retrieval from LDs has been reduced with the development of surgical methods. For example, in a trial performed by a Chinese team, the procurement surgery of the uterus with complete robot assistance lasted for 6 hr (25). Additionally, in an Indian trial using traditional laparoscopy with laparotomy, the authors have reported this time as approximately 3-4 hr (27, 28).

In LD, the length of the excised vascular pedicles is shorter, and the risk of damage to them is higher than in DD (58). In the uterus retrieval from DD, it is possible to harvest longer vascular pedicles with a larger diameter (4, 11, 17, 52), which will lead to better and easier suturing of the vascular pedicles, a lower risk of thrombosis in the transplanted organ (4, 52), and reduced complications and rejection rate in the recipient (12, 52).

In DD, it is possible to cut the ureters on both sides of the uterine vascular pedicles; nevertheless, in LD, it is necessary to meticulously examine the ureteric tunnels to separate the vessels from the ureters (8) as deep uterus veins are firmly attached to the ureters (3). The most sensitive part of the LD hysterectomy is the 360º dissection of the ureteric tunnel when releasing the uterine vascular pedicle (34). However, in DD, a major part of the dissection of the uterine vein can be conducted by separating branches from the deep iliac vein and from the uterovesical venous plexus by a bipolar sealing device to reduce the risk of damage to the adjacent tissues (8). As the accurate identification and separation of uterine vessels, particularly veins, is quite challenging, it is possible in DDs to use ovarian vessels for vascular anastomosis; nonetheless, in premenopausal LDs, there is no desire for oophorectomy, using them is not possible (11). Moreover, in DDs, the excision of a more extended vaginal cuff for a better vaginal-to-vaginal anastomosis and, thus, the elongation of the recipient's vagina is possible (59).

### Implementation of the transplantation procedure

#### Logistics of surgical procedure 

For the UTx procedure, one surgical team for the retrieval, one team for preparation of the graft on the back table, one team for transplantation, and 2 anesthesia care teams, one for the donor and one for the recipient, are usually needed. Each surgery team includes at least 2 or 3 members (4). The procedure can be preplanned with the entire multidisciplinary team. This approach allows all key members to rest well and prepare appropriately (54). Unaccelerated preparation can lead to learning and memorization of the various stages of the surgery (4). In addition, having the same surgical team is an advantage that leads to faster learning of different parts of the procedure. In planned surgery in LD, a roadmap can be developed before transplantation, which can secure a shorter and safer surgery (8).

When a DD is identified, the above-mentioned 4 or 5 teams should be prepared quickly; given that brain death cannot be anticipated, these teams should be prepared 24 hr a day and 7 days a week. Surgery might be more difficult due to the need for rapid retrieval of the organ. Less time for preparation with excessive fatigue and stress associated with difficult procedures, possibly performed at night, can increase surgery complications (4). On the other hand, in the circumstances that the donation is carried out in a different hospital that is far from the transplant hospital, if the same surgeons perform the transplantation, the workload of the uterus-harvesting team might significantly increase, and this can negatively affect the transplantation procedure (8). Logistics, such as air transportation on permanent standby in deceased donation, are also required (4).

#### Waiting time

Considering the shortage of DDs (52, 58), the waiting time for receiving a matched uterus graft from a DD is uncertain. The recipients must be on continuous standby, and this might lead to the frustration of transplant candidates or the demobilization of the medical team in the experimental phase (4). Therefore, the benefit of using an LD is the shorter waiting time for the transplantation (10).

#### Costs of transplantation

As using DD requires logistical measures, such as air transportation on permanent standby, on-call medical teams (4), or access to an operating room for a relatively long surgery (59), presumably, DD UTx will be more expensive than LD UTx (4). Nonetheless, no donor recovery and postoperative costs are among the advantages of using DD (29).

### Outcomes of transplantation 

#### Graft failure

UTx failure is defined as the need to remove the graft before embryo transfer (60). According to the data in table I, the graft failure rates in LD, and DD UTx procedures are 21% (11/52) and 36% (4/11), respectively. Given that out of 63 described procedures, almost 82.5% and 17.5% were LD and DD UTx, respectively, comparing the failure rate between the 2 approaches to donation is difficult and demands more DD UTx procedures.

Several studies have demonstrated that graft failure can be partially attributed to the poor quality of the graft, particularly its poor perfusion (3). After menopause, the size of the uterus decreases, periodic fluctuations in the uterus blood flow are no longer present (8), and age-related negative changes in the myometrium, endometrium, and or uterine vasculature might also occur (3). On the other hand, atherosclerosis develops with the increase in age. Therefore, the older age of the donor can be an obstacle in using the organ of both LD and DD (8). LDs are typically older than DDs (52) because, in living donation, a woman who has completed her parity and no longer needs the reproductive function of the uterus can be a candidate for uterine donation (4).

Another factor that negatively affects the organ's quality is systemic inflammation observed in DDs (4, 17, 18, 47, 48). Ischemia can also negatively affect the quality of the graft (4). Ischemia at body temperature (i.e., warm ischemia [WI]) or during the storage of the graft outside the body at hypothermic condition (i.e., cold ischemia [CI]) might lead to histologic and metabolic damages, which are intensified during the reperfusion of the organ and lead to acute or chronic loss of the organ functions (60).

The CI time in DDs is usually longer than in LDs because the uterus of an LD is transmitted to a recipient in a near operating room; however, the uterus of a DD might be transmitted to a recipient in another center (17). Furthermore, back-table evaluation increases the CI time (8). Nonetheless, several studies have demonstrated that the uterus has good tolerance to CI (40). For example, in deceased donation, long periods of CI up to 6 hr and 20 min (16) or 9 hr and 9 min (52) have led to live birth. The WI occurs in 2 stages; the first stage of WI occurs at the time of organ harvesting (48). The second stage occurs during vascular anastomosis in the recipient (60). The longer harvesting time leads to a higher risk of damage to the organ (48). In DD, harvesting the nonvital organs, such as the uterus, should be after the vital ones, and this can increase the time of the first stage of WI (40).

Regarding the long-term survival of the graft, Kisu et al. believe that in deceased donation, due to organ damage as a result of factors, including prescription of catecholamines to the donors in the agonal stage, systemic inflammatory changes, and prolonged ischemia time (40), long-term graft survival is less than a living donation (48). Nonetheless, this is not of great importance for women whose uterus is removed after the delivery, as UTx is considered temporary and short-term transplantation (40).

Another factor that influences graft survival is histocompatibility. The higher human leukocyte antigen (HLA) compatibility leads to better graft survival outcomes (48). Using intrafamilial LDs' uterine graft reduces complications related to histocompatibility (4) and exposure to foreign HLA (8). If the donor is the recipient's mother, they match at least half of the HLAs; if she is her sister, the HLA complete matches are 25% (48). In deceased donation, the histocompatibility between the donor and the recipient is low, and there is a greater risk of graft rejection. Therefore, the recipient needs higher doses of immunosuppressive drugs, which increases the risk of oncologic and infectious side effects (4).

#### Live birth 

The primary goal of UTx is a live birth (61). According to the data of table I, supposing that all recipients who did not have graft failure underwent embryo transfer, live birth rates in LD and DD UTx procedures are almost 63% (26/41) and 71% (5/7), respectively. Although the risk of pregnancy complications after solid organ transplantation and in vitro fertilization is significantly higher than spontaneous pregnancy without a transplant, there might be differences in the incidence of these complications between the 2 donation methods. For example, regarding the risk of preeclampsia during pregnancy following UTx, it has been suggested that sclerotic and low elastic arteries in older LDs can increase the risk (52). Nevertheless, in young DDs, there is no vascular pathology, such as atheroma, and arteries have elastic walls, which can prevent obstetrics complications, such as preeclampsia (4).

In DD uterus recipients, pregnancy complications, such as placenta previa with accrete (29), pyelonephritis (16), preterm premature rupture of the membranes, intrauterine growth restriction, suspected preeclampsia (20), and gestational diabetes mellitus (35) have been reported. Intrahepatic cholestasis (21), pre-eclampsia, hydronephrosis (15), placenta previa marginalis, pregnancy-associated hypertension (35), anemia (21, 34), gestational diabetes (34, 35), vaginal bleeding (24, 32, 34), cervical incompetence, preterm delivery, intrauterine fetal demise (34), preterm prelabor rupture of membranes, mild oligohydramnios, and small for gestational age (30) are also documented as pregnancy complications in LD uterus recipients. As in comparison to LD UTx procedures, only a small number of DD UTx procedures have been conducted, a complete comparison of the rates of live births and pregnancy complications between these 2 types of donation needs further data.

Collecting more data on the effectiveness and safety of using the uterus of DDs makes it possible to accurately compare the outcomes and risks of living and deceased donation (29). If the long-term outcomes of deceased and living donation are similar, or even if deceased donation outperforms living donation (53, 62), and there are enough uteri from DDs available, living donation will not be continued to avoid risks for LDs (6, 13, 53). Nonetheless, considering the expected increase in demand for UTx in the future and the shortage of uterine grafts (4, 58), currently, UTx through both types of donation is rational.

##  Strengths and limitation 

In the present study, the articles were searched with no time, location, or language limitations, which is one of the study's strengths. The clarity of the search strategy also makes it possible for other researchers to replicate it. Moreover, this review study only includes the articles on the PubMed database.

##  Implication and suggestions

The current study findings can be informative for both clinicians and the public. Considering the experimental nature and the relatively small number of UTx procedures performed worldwide, I recommend implementing further procedures, especially DD UTx. I also suggest making great efforts to raise public awareness about UTx and methods of uterus donation, particularly deceased donation.

## 4. Conclusion

Both living and deceased donations can provide suitable uteri for delivering a healthy neonate. The main disadvantage of using LD is the risks for the donor. In the deceased donation, there is no such concern; however, restricted uterus assessment before donation and the lack of the possibility for planned surgery are the main disadvantages of using the uterus of DD. Given the insufficient available data, it is difficult to compare the clinical outcomes between the 2 types of donation. Therefore, it is rational to use a combination of both LD and DD at this stage.

##  Conflict of Interest

The author declares that she has no conflict of interest in the present study.
